# Atrial fibrillation in the Indigenous populations of Australia, Canada, New Zealand, and the United States: a systematic scoping review

**DOI:** 10.1186/s12872-015-0081-6

**Published:** 2015-08-13

**Authors:** Judith M. Katzenellenbogen, John A. Woods, Tiew-Hwa Katherine Teng, Sandra C. Thompson

**Affiliations:** Western Australian Centre for Rural Health, The University of Western Australia (M706), 35 Stirling Highway, Crawley, Western Australia 6009 Australia; School of Population Health, The University of Western Australia (M431), 35 Stirling Highway, Crawley, Western Australia 6009 Australia

## Abstract

**Background:**

The epidemiology of atrial fibrillation (AF) among Indigenous minorities in affluent countries is poorly delineated, despite the high cardiovascular disease burden in these populations. We undertook a systematic scoping review examining the epidemiology of AF in the Indigenous populations of Australia, Canada, New Zealand (NZ) and the United States (US).

**Methods:**

PubMed, Scopus, EMBASE and CINAHL-Plus databases were systematically searched in May 2014. Supplementary full-text searches of Google Scholar and government website searches were also undertaken.

**Results:**

Key findings from 27 publications with diverse aims and methods were included. Small studies from Canada and NZ suggest higher AF prevalence in Indigenous than other populations. However, this was not reflected in a large sample of US male military veterans. No data were identified on community-based incidence rates of AF in Indigenous populations. Australian and Canadian studies indicate higher first-ever and overall AF hospitalisation rates among Indigenous than other populations, at younger ages and with more comorbidity. Studies in stroke, heart failure and other clinical groups demonstrate AF as a common comorbidity, with AF possibly more prevalent at younger ages in Indigenous people. Indigenous patients have similar early post-hospitalisation adjusted mortality but higher 1-year risk-adjusted mortality than non-Indigenous patients.

**Conclusions:**

No clear epidemiological pattern of AF frequency across the considered Indigenous populations emerges from the limited available evidence. AF should be included in key conditions reported in national surveillance reports, although Indigenous identifiers are required in administrative data from Canada and the US. Sufficiently powered, community-based studies of AF epidemiology in diverse Indigenous populations are needed.

**Electronic supplementary material:**

The online version of this article (doi:10.1186/s12872-015-0081-6) contains supplementary material, which is available to authorized users.

## Background

Indigenous peoples living in affluent countries have poor health profiles and considerably diminished life expectancies compared with their non-Indigenous counterparts [1–3]. Although the Indigenous populations of Australia, Canada, New Zealand (NZ) and the United States (US) are highly diverse [4], they ‘share similar historical experiences, socioeconomic disadvantage, and health status’ [5]. As such, these four populations ‘are often seen as natural comparators in terms of Indigenous wellbeing’ [6] in health research literature [7–9] and elsewhere [10]. An excess burden of cardiovascular diseases (CVD), typically presenting at a younger age, predominates the gap in their health outcomes [11, 12].

Atrial fibrillation (AF), the most common sustained cardiac arrhythmia, is increasing in prevalence and incidence globally [13–15]. Prevalence increases progressively with age, and is higher among men than women [16]. AF causes serious complications, notably heart failure and thromboembolic sequelae such as stroke [17–19], with stroke prevention a cornerstone of management [20]. AF is associated with a substantial increase in overall mortality [21], although the direct causality of this association remains contentious [22].

AF characteristically accompanies the spectrum of common CVDs disproportionately afflicting Indigenous populations [11, 23], so this arrhythmia could be predicted to affect them with increased frequency. However, many large studies and recent international reviews of AF epidemiology notably lack data on Indigenous populations [13–15, 24]. This omission may be related to the low proportion of Indigenous people in most jurisdictions, and to inadequate documentation of Indigenous identity in administrative data. This paper reports a scoping review mapping the current knowledge of AF epidemiology in the Indigenous populations of Australia, Canada, NZ and the US.

## Methods

### Information sources and search strategy

Indigenous populations considered were those of Australia (Aboriginal and Torres Strait Islander peoples), NZ (Māori), Canada (Aboriginal peoples comprising First Nations, Métis, and Inuit), and the US (Native American, Native Alaskan, and Native Hawaiian).

The multifaceted search strategy incorporated both journal publications and ‘grey’ literature (including conference abstracts). An electronic database search incorporating PubMed, Scopus, EMBASE and CINAHL-Plus was conducted in January 2014 and updated in May 2014. Records retrieved were those containing terms related to both Indigenous populations and atrial fibrillation (Additional file 1: Table S1), with an equivalent search conducted in each database. To maximise identification of peer-reviewed papers, the standard multi-database search was supplemented by a series of full-text journal article searches using Google Scholar. In view of the limited Boolean searching functionality of Google, a series of complementary simple searches was done to maximise identification of pertinent references. In these searches (23/05/2014), using the ‘Sort by relevance’ function, the phrase-forced term “atrial fibrillation” was searched separately in combination with each of the following terms: aboriginal, maori, “native american”, “first nations”, metis, inuit and indigenous. The full-text of the first 50 references retrieved (or all references if <50 were retrieved) in each of these searches was reviewed, except that in the final search (“indigenous” and “atrial fibrillation”), the first 100 references were reviewed. A second series of Google scholar searches date-restricted to 2014 was also done (27/05/2014), to maximise retrieval of recent publications that may have been missed in the systematic multi-database search.

The grey literature was searched using Google as well as applicable government departmental websites from each country. Additionally, advice was sought on data sources that may have been overlooked, by means of direct email contact with relevant experts in NZ and Canada, in order to complement the authors’ pre-existing knowledge of Australian administrative health data collections.

The systematic searches were supplemented by citation screening of retrieved records and the addition of publications otherwise known to the authors.

### Study selection and inclusion criteria

Records retrieved from the searches were catalogued in EndNote®. Duplicates were removed by automation supplemented with manual checking.

Publications considered for inclusion were those containing original epidemiologic or health services data on AF in the designated Indigenous populations. ‘Epidemiological data’ was broadly defined to include metrics such as incidence, prevalence, aetiology, risk factors and health outcomes (including mortality). Inclusion was date-restricted to articles published from 1980 onwards. There was no formal restriction by language of publication. However, all pertinent records identified had been published in English or bilingually.

Full-length articles as well as abstracts (where no full length published article was identified) were eligible for inclusion. One reviewer (JAW) excluded studies judged on the basis of title and abstract to be clearly non-relevant according to pre-defined criteria (pre-1980; wrong country; case report; non-anthropological meaning of ‘indigenous’ such as indigenous botanical). Two reviewers (JMK and JAW) each screened the remaining titles and abstracts for relevance. Disagreements were resolved by consensus. Articles were excluded if Indigenous data were only included as baseline characteristics or had been combined with other minority ethnic groups, or if there were few (<10) Indigenous AF cases.

### Data extraction

Article details were extracted independently by reviewers (JMK, T-HT, JAW) onto a predesigned spreadsheet template. Studies were grouped according to the type(s) of epidemiological data on AF presented. The validity of study hypotheses on epidemiological indices of Indigenous AF was assessed in applicable cases using the Newcastle-Ottawa Scale (NOS), which awards up to 9 or 10 stars for quality, based upon assessment of sample selection and comparability, and exposure/outcome ascertainment [25]. Papers were categorised by type of epidemiological data provided.

## Results

**Fig. 1:**
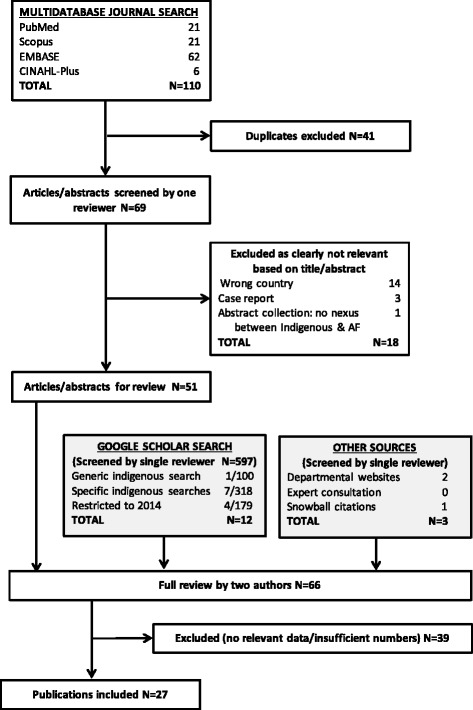
Flowchart of search strategy and output

Of 69 unique publications identified, 51 went to full review, of which 27 met the inclusion criteria (Fig. 1). The US and Australia contributed the majority, with descriptions of AF in clinical groups (50 %) and recent publications (2006 onwards) dominating (Table 1). In the majority of papers identified, either AF was incidental to the study rather than a core or major focus, or Indigenous data were incidental or uninterpretable due to insufficient subject numbers. In the interval between the database searches and manuscript completion, data from two of the identified abstracts [26, 27] and one report [28] were published as peer-reviewed journal articles and were updated as such in the review [29–31].

**Table 1:** Characteristics of publications retrieved—classified by country

	Australia	New Zealand	USA	Canada	Total
Publication type					
Journal article	8	2	7	3	20
Conference abstract	0	3	2	0	5
Report	1	1	0	0	2
Study design					
Cohort	1	1	2	1	5
Case–control	0	0	0	0	0
Cross-sectional	4	0	5	0	9
Descriptive	4	5	2	2	13
Epidemiological index or theme^a^					
Antecedents of AF	0	0	2	0	2
Incidence of AF in a population	1	0	0	1	2
Prevalence of AF in a population	1	2	1	1	5
AF in primary care consultations	1	0	0	0	1
AF hospital admission rates	0	1	0	0	1
Outcomes in AF patients	1	1	1	1	4
Health service provision	0	0	0	1	1
AF as an outcome	0	0	1	0	1
Occurrence of AF in a clinical group	6	2	4	2	14
Primary focus on AF					
Yes—Indigenous AF	3	2	3	1	9
Yes—AF (Other)	0	1	3	0	4
No	6	3	3	2	14
Setting					
Community	1	2	4	0	7
Primary care	1	0	0	0	1
Hospital patients: no population denominator	4	3	4	1	12
Hospital patients: population denominator	3	1	0	2	6
Hospital patients and community	0	0	1	0	1
Calendar period of final data collection					
1980-1995	0	1	0	0	1
1996-2005	0	2	3	0	5
2006 onwards	9	3	6	3	21
Total	9	6	10	3	27

^a^Studies may be included in more than one category

### Antecedents of AF

No studies were identified with data on the independent relationship of AF to antecedent lifestyle risk factors or medical conditions among Indigenous populations, or on Indigenous versus non-Indigenous differentials in this regard. The Strong Heart Study [11], a large US cohort restricted to Native American subjects followed up for 10 years, produced two abstracts investigating novel AF antecedents in this population (Table 2). Independent risk factors for new-onset AF were increasing age (HR = 1.08), male sex (HR = 1.67), C-reactive protein (HR = 1.44) [32], serum fibrinogen (HR = 1.31) [32], Left Ventricular Mass Index (HR = 1.31) [33], and Left ventricular ejection fraction (HR = 0.73) [33].

**Table 2:** Studies of antecedents and population-based occurrence of atrial fibrillation

Author (Year) Publication type	Country Indigenous population Calendar period	Methods	Key findings on Indigenous AF	Quality score (Newcastle-Ottawa Scale applied only to Indigenous AF data) Comments
Antecedents of AF				
*Title: Association of Markers of Inflammation with New-Onset Atrial Fibrillation in a Population-Based Sample: The Strong Heart Study*				
Zacks (2006) [32] Conference abstract	Country: US	Design: Population-based cohort study	New-onset AF (n = 100 participants) independently predicted by serum CRP level (HR 1.44 per mg/L [95 % CI 1.17–1.77], *p* = 0.001), and by fibrinogen level (HR 1.31 per 83.44 mg/dL [=1 SD of mean][95 % CI 1.06–1.61], *p* = 0.013)	NOS: N/A (abstract) No non-American Indian comparison group; data presented as generalisable evidence that CRP & fibrinogen are additive risk factors for new-onset AF (independent of effects of gender, age, hypertension, BMI, and urinary albumin-creatinine ratio)
	Population: American Indians	Data Source: Strong Heart		
	Period: enrolled 1993–1995 with 10 years follow-up	Study: prospectively collected population-based survey of risk factors		
	Sample size: 3541	Setting: 13 American Indian communities		
		Sample size: 3541		
*Title: Association of Left Ventricular Mass and Ejection Fraction with New-Onset Atrial Fibrillation in a Population-Based Sample: The Strong Heart Study*				
Zacks (2006) [33] Conference abstract	Country: US	Design: Population-based cohort study	New-onset AF (n = 91 participants) independently predicted by increased LV mass indexed for height (HR 1.49 per 11 gm/m^2.7^ [=1 SD of mean][95 % CI 1.24–1.78], p ≤ 0.0001), and (n = 88) by reduced LVEF (HR 0.65 per 14 % [=1 SD of mean][95 % CI 0.52–0.82], p ≤ 0.0001)	NOS: N/A (abstract) No non-American Indian comparison group; data presented as generalisable evidence that LV mass index and LVEF are additive risk factors for new-onset AF (independent of effects of gender, age, hypertension, BMI, urinary albumin-creatinine ratio, CRP and fibrinogen)
	Population: American Indians	Data Source: Strong Heart		
	Period: enrolled 1993–1995 with 10 years follow-up	Study: prospectively collected population-based survey of risk factors		
	Sample size: 3541	Setting: 13 American Indian communities		
		Sample size: 3541		
Incidence in population				
*Title: Cardiovascular Disease Rates, Outcomes, and Quality of Care in Ontario Métis: A Population-Based Cohort Study*				
Atzema (2015) [31] Journal article (this study has multiple outcomes)	Country: Canada (Ontario only)	Design: Retrospective cohort study (18 % of Métis population)	Age- & sex-adjusted incidence per 100 (CI): Métis0.62 (0.50–0.73)	NOS (cohort): 7/9 Incidence well-defined. Register not representative; Out-of-hospital cases not included; very small numbers of incident cases
	Population: Métis	Data Source: Ontario Métis register linked to emergency department (ED), in-patient hospital & mortality records	All Ontario 0.32 (0.32–0.32)	
	Period: 2006-2011	Setting: ED and hospital based cases	*p* < 0.001	
	Age: 20 years & over	Other: 5-year clearance period		
		Sample size: 56 cases of 12,550 (7 % of provincial Métis population)		
*Title: Initial hospitalisation for atrial fibrillation in Aboriginal and non-Aboriginal populations in Western Australia*				
Katzenellenbogen (2015) [30] Conference abstract later published as a journal article (this study has multiple outcomes)	Country: Australia (Western Australia only)	Design: baseline data of retrospective cohort	Aboriginal age-specific rates higher than non-Aboriginal rates in all ages <70 years	NOS (adapted for cross-sectional): 10/10 Coverage of whole State with linked data but admitted hospital cases only; no data on diagnostic tests and medications; diagnostic codes not validated
	Population: Aboriginal	Data Source: Linked hospital and death records	ASRR: 20–54 years = 3.6 (males) and 6.4 (females) 55–84 years = 1.3 (males) and 1.8 (females)	
	Age: 20–84 years	Setting: Western Australian hospital cases		
	Period: 2000-09	Other: 15-year clearance period		
		Sample size: 37,097 AF cases, 923 Aboriginal		
Prevalence in population				
*Title: Cardiovascular Disease Rates, Outcomes, and Quality of Care in Ontario Métis: A Population-Based Cohort Study*				
Atzema (2015) [31] Journal article (this study has multiple outcomes)	Country: Canada (Ontario only)	Design: Retrospective study	Age- & sex-adjusted prevalence per 100 (CI): Métis 2.08 (1.82–2.34)	NOS (adapted for cross-sectional): 8/10Prevalence not well-defined. Register not representative, out-of-hospital cases not included, numerators not provided and likely to be small numbers
	Population: Métis	Data Source: Métis register linked to emergency department (ED), in-patient hospital & mortality records	All Ontario 1.42 (1.41–1.43)	
	Period: 2006-2011	Setting: ED and hospital based cases	*p* < 0.001	
	Age: 20 years & over	Sample size: 12,550 (17 % of provincial Métis population)		
*Title: Racial differences in the prevalence of atrial fibrillation among males*				
Borzecki (2008) [34] Journal article	Country: US	Design: Cross-sectional	Prevalence in male Veterans higher among White than Native Americans Age-adjusted: White 5.7 % Native American 5.4 % Multivariate OR 1.15; 95 % CI 1.04-1.27 (adjusted for age, BMI and predisposing comorbidities)	NOS: (adapted for cross-sectional) 10/10 High quality whole-of-nation study. Survey response only 67 % Whites & 55 % Native Americans, but analyses of administrative data from non-respondents support lower prevalence of AF among Native Americans vs Whites. Restricted to male veterans: military recruiting may limit generalisability
	Population: Native American/Alaskan/Hawaiian	Data Source: administrative database plus health survey		
	Period: 1997-1999	Setting: population-based (male veterans)		
	Age: 18 years & over	Sample size: 664,754 respondents (27,697 Native Americans)		
*Titles: 1. Heart failure, ventricular dysfunction and risk factor prevalence in Australian Aboriginal peoples: the Heart of the Heart Study*				
*2. Cardiometabolic risk and disease in Indigenous Australians: the Heart of the Heart Study*				
McGrady (2012) [36] Brown (2014) [35] Journal articles	Country: Australia	Design: Cross-sectional	Crude prevalence of AF = 2.5 % Similar prevalence <40 and 40–55 years (1 %; n = 3), higher prevalence 56+ years (8 %; n = 8). Similar prevalence between remote and town communities.	NOS (adapted for cross sectional): 8/10 (AF not main outcome) Standardised measurements; out-of-hospital and undiagnosed cases included; small numbers; estimated 10 % enrolled, representativeness unknown, possible selection bias
	Population: Aboriginal	Data Source: Community survey, including psycho-social, biological and clinical measures		
	Age: 17+ years	Setting: 3 communities in Central Australia		
	Period: 2008-09	Sample size: 436 volunteers		
*Title: Twelve Lead Electrocardiographic Findings Among Māori and non-Māori at Risk of Cardiovascular Disease in NZ*				
Martin (2013) [37] Conference abstract	Country: NZ	Design: baseline descriptive (within cohort study)	Atrial fibrillation frequencies: 2 % rural Māori 1.2 % urban Māori 0.4 % urban non-Māori	NOS: N/A (abstract) No data provided on age/sex distribution, no statistical inference
	Population: Māori	Data Source: ‘randomly selected’ community samples from the Hauora Manawa Community Heart Study cohort: 12-lead ECG		
	Age: 20–64 years	Setting: two Māori Communities (rural, urban) and a non-Māori urban cohort		
	Period: Not known	Sample size: 252 rural Māori, 243 urban Māori, 256 urban non- Māori		
*Title: The Burden of Atrial Fibrillation in Octogenarians*				
Teh (2013) [38] Conference abstract	Country: NZ	Design: baseline descriptive (within cohort study)	30 % Māori versus 21 % non-Māori had AF, either on ECG or NZHIS records 7 % Māori versus 4 % non-Māori had AF newly detected by study ECG	NOS: N/A (abstract) No statistical inferential data or eligibility exclusions reported Stroke reported as a comorbidity in 27 % of Māori and 35 % of non-Māori subjects
	Population: Māori	Data Source: Life and Living to Advanced Age (NZ) cohort: 12-lead ECG plus NZHIS		
	Age: 80-90	Setting: community		
	Period: 2010-2011	Sample size: Overall cohort: 421 Māori aged 80–90; 516 non- Māori all aged 85.615 (66 %) participants had ECG; 870 (93 %) consented to NZHIS record examination		
Admission Rates (unlinked)				
*Title: The Management of People with Atrial Fibrillation and Flutter: Evidence-based Best Practice Guideline*				
New Zealand Guidelines Group (2005) [39] Report	Country: NZ	Design: Descriptive	Hospital discharges with AF diagnosis: Age-standardised rate for Māori almost twice that of non-Māori (104 per 100,000 vs 57 per 100,000, *p* < 0.05) Standardised discharge ratio (observed versus expected) 1.945 for Māori & 0.972 for ‘others’ (where 1.0 is the national average) Modal age group: Māori 65–69 years, ‘other’ males 75–79 years, ‘other’ females >85 years	NOS: N/A (report with insufficient methodological detail published) Unlinked administrative data
	Population: Māori	Data Source: National minimum dataset		
	Period: 2001-2002	Setting: Hospital patients		
	Age: unrestricted	Sample size: (whole of NZ data; sample size not stated)		

*AF* atrial fibrillation, *US* United States, *NOS* Newcastle-Ottawa Scale, *N/A* not applicable, *CRP* C-reactive protein, *HR* hazard ratio, *CI* confidence interval, *SD* standard deviation, *BMI* Body-mass index, *LV* left ventricle, *LVEF* left ventricular ejection fraction, *ED* emergency department, *ASRR* age-standardised rate ratio, *OR* odds ratio, *NZ* New Zealand, *ECG* electrocardiograph, *HZHIS* New Zealand Health Information Service

### Population-based epidemiological indices of AF

#### Incidence

Two studies provided data on population-based AF hospitalised incidence among Indigenous peoples (Table 2). A Canadian study provided cohort data from the Ontario Métis Register linked with emergency department and hospital inpatient records. The age- and sex-adjusted incidence of AF per 100 persons (aged 20–105) was 0.62 (95 % confidence interval [CI] 0.50–0.73) in the Métis versus 0.32 (CI 0.32–0.32) in the general Ontario population (*p* < 0.001) [31]. Individuals identified in the register constituted only 18 % of Ontarian Métis, and may not have been representative of the provincial Métis population. Additionally, the number of incident AF cases was very small (n = 56). A Western Australian (WA) study using linked inpatient and mortality records provided estimates for different age and sex groups, highlighting the greater disparities in younger adults [30]. At all age groups under 65 years, Aboriginal rates were significantly higher than non-Aboriginal rates. Among adults aged 20–54 years, the age-standardised rate ratios (ASRRs) for incident (hospitalised) AF were 3.6 in males and 6.4 in females; in the 55–84 year age group, ASRRs were 1.3 and 1.8 respectively. AF was more likely in Aboriginal than among non-Aboriginal people to be an emergency admission and a diagnosis secondary to another principal diagnosis (mainly other CVD). Additionally, case complexity as reflected in co-morbidity profile was greater in Aboriginal patients. Both publications relied on hospital data only, thus not capturing incident cases in the community.

#### Prevalence

Among the studies that investigated AF prevalence in Indigenous versus other racial groups (Table 2), only one (from the US) provided whole-of-nation comparative data [34]. In this large study (total subjects n = 664,754; Native American n = 27,697), based on data linked between two national Veterans Health Administration administrative databases and a mailed questionnaire survey, the age-adjusted prevalence of AF was similar among White (5.7 %) and Native American (5.4 %) males. Subjects included in these estimates were those who responded to the survey (response 67 % Whites, 55 % Native Americans). After multivariate adjustment for age, body-mass index (BMI) and predisposing comorbidities, AF was less prevalent among Native American than White adult males (adjusted odds ratio White versus Native Americans: 1.15; CI 1.04-1.27).

The Ontario Métis Register provided data derived from emergency department (ED) and hospital databases on AF prevalence among Ontarians [31]. The reported age- and sex-adjusted prevalence of AF in subjects 20 years and over was 2.08 (CI 1.82–2.34) per 100 persons in the Métis, versus 1.42 (CI 1.41–1.43) in the general Ontarian population (*p* <0.001). However, these data are difficult to interpret as the basis for measuring prevalence was not clearly defined, and information on out-of-hospital cases was lacking.

Two peer-reviewed journal papers based on the Heart of the Heart Study [35, 36] reported data on AF prevalence in three central Australian Indigenous communities, providing no comparative data. This cross-sectional study was designed to investigate the burden and correlates of cardiovascular and other diseases in a representative sample of community dwelling adults. The study incorporated detailed clinical and diagnostic cardiovascular assessment of 436 volunteer subjects. The crude reported prevalence of AF was 2.5 %; this predictably rose with age. Caveats on the interpretation of these estimates include the small number of subjects and the unknown representativeness of the sample in relation to the communities.

Prevalence data of AF from separate cohort studies among Indigenous (Māori) communities from NZ were reported in two conference abstracts. Firstly, in data from the Hauora Manawa Project, randomly selected community-based samples, each comprising about 250 individuals from two Māori Communities (one rural and one urban), were compared with an urban non-Māori community [37]. The reported crude AF prevalence was higher in both Māori communities (2 % and 1.2 % respectively) than among the non-Māori subjects (0.4 %). The other abstract reported AF frequency among 937 octagenarian subjects (421 Māori, 516 non- Māori) [38]. AF was more common among Māori (30 %) than non-Māori participants (21 %).

#### Life-time risk

No publications provided data on life-time risk of AF among the Indigenous populations of the selected countries.

#### Hospital admission rates

No peer-reviewed publications were identified with population-based data on AF-related overall hospital admission frequency according to Indigenous status. The only whole-jurisdictional administrative data on Indigenous AF admissions were those reported from the NZ National Minimum Dataset where the age-standardised rate of hospital discharges in 2001–02 among Māori was almost twice that among non-Māori (104 per 100,000 vs 57 per 100,000, *p* < 0.05) [39]. These data were unlinked, precluding person-level analysis of admission patterns. Publications from the Australian Institute of Health and Welfare report unlinked data on hospitalisations for CVD and do not distinguish AF from other cardiac conditions in relation to Indigenous hospitalisations [40].

### Outcomes in AF patients

Three publications, two from North America and one from Australia, provided data on outcomes among Indigenous patients hospitalised with AF (Table 3). In the study on CVDs among the Métis Nation of Ontario, age- and sex-standardised all-cause mortality (n = 6 Métis deaths) at one year following incident AF admission or emergency presentation was 2.1 times higher among Métis than the general Ontarian population (*p* = 0.06; borderline significance). There was no significant difference in disease-specific hospitalisations in the first year after incident diagnosis (rates age- and sex-standardised to those of general Ontarian population 1.23 (CI 0.73-2.08, *p* = 0.44) [31]. A US study based on the 2008 Nationwide Inpatient Sample, which provides data on all discharges from approximately 1000 hospitals, investigated in-hospital mortality of patients with a primary discharge diagnosis of AF [41]. Native Americans accounted for 0.75 % of the sample of 425,470 hospitalisations. The odds ratio for in-hospital mortality following admissions of Native American versus White, adjusted for age, sex and comorbidities, was 0.732 (CI 0.227–2.358, *p* = 0.30). Limitations of the database precluded a person-based analysis that could track readmissions.

**Table 3:** Studies of atrial fibrillation outcomes

Author (Year) Publication type	Country Indigenous population Calendar period Age range	Methods	Key findings on Indigenous AF	Quality score (Newcastle-Ottawa Scale applied only to Indigenous AF data) Comments
Outcomes in AF patients				
*Title: Cardiovascular Disease Rates, Outcomes, and Quality of Care in Ontario Métis: A Population-Based Cohort Study*				
Atzema (2015) [31] Journal article (this study has multiple outcomes)	Country: Canada (Ontario only)	Design: Retrospective cohort study	Age- & sex-adjusted all cause mortality (CI) Métis 16.6 (7.3–25.4) All Ontario 7.8 (7.5–8.1) *p* = 0.06	NOS (cohort): 7/9 ‘Incidence case’ denominator determined by first emergency department presentation or hospitalisation onlySmall number of Métis subjects
	Population: Métis	Outcomes: One-year all-cause and cardiovascular mortality in incident cases ()	Age- & sex-adjusted cardiovascular mortality (CI) Métis 10.0 (2.4–17.7)	
	Period: 2006-2011	Sample size: 6 deaths in 56 Métis; 32,387 general Ontarian incident cases	All Ontario 4.8 (4.6–5.0) *p* = 0.19	
	Age: 20+ years			
*Title: African Americans have the highest risk of in-hospital mortality with atrial fibrillation related hospitalizations among all racial/ethnic groups: A nationwide analysis*				
Turagam (2012) [41] Journal research letter	Country: US	Design: cross-sectional/cohort	In-hospital mortality following admission with AF as principal diagnosis: Native Americans vs Whites adjusted HR 0.7 (*p* = 0.3)	NOS (adapted for cross sectional) 8/10 Unlinked data; short follow-up (hospital deaths only)
	Population: Native American	Data Source: Nationwide Inpatient Sample hospitalization database		
	Period: 2008	Setting: hospitals		
	Age: uncertain	Sample size: 425470 admitted with AF as principal diagnosis		
*Title: Initial hospitalisation for atrial fibrillation in Aboriginal and non-Aboriginal populations in Western Australia*				
Katzenellenbogen (2015) [30] Conference abstract later published as a journal article (this study has multiple outcomes)	Country: Australia (Western Australia only)	Design: Retrospective cohort	1-year mortality: cross-over effect 30-day mortality: Demography-adjusted HR = 1.7	NOS (cohort): 9/9 Hospitalised cases only AF codes not validated No diagnostic tests and therapeutic data
	Population: Aboriginal	Data Source: Linked hospital and death records	Fully adjusted HR = 0.81 (NS) 1-yr mortality in 30-day survivors: Demography-adjusted HR = 2.9	
	Age: 20–84 years	Setting: Western Australian hospital cases	Fully adjusted HR = 1.6 Comorbidities impact substantially on attenuation of effect	
	Period: 2000-09	Other: 15-year clearance period		
		Sample size: 37,097 AF cases, 923 Aboriginal; 5,417 mortality events		
AF as an outcome				
*Title: Race/ethnicity and the incidence of new-onset atrial fibrillation after isolated coronary artery bypass surgery*				
Nazeri (2010) [42] Journal article	Country: US	Design: retrospective cohort	Cumulative incidence prior to discharge of new-onset post-operative AF (crude percentages; no statistical inference) Caucasians: 32.4 % Native Americans: 18.8 %	NOS (cohort) 7/9 Descriptive study only in relation to Native Americans Very small number of Native Americans insufficient for multivariate analysis
	Pop: Native Americans	Data Source: Institutional research database		
	Period: 2000-2008	Setting: Single tertiary hospital		
		Sample size: Total: 5823		
		Native American: 11 (0.2 %)		

*NOS* Newcastle-Ottawa Scale, *US* United States, *AF* atrial fibrillation, *HR* hazard ratio

In the Western Australian study based on linked hospitalisation and mortality records [30], there was a cross-over of early survival, with 30-day adjusted mortality tending to be lower (not significant) in Aboriginal than non-Aboriginal patients in their first-ever AF admission. However, the adjusted hazard ratio for one-year mortality in Aboriginal versus non-Aboriginal 30-day survivors was 1.58. Comorbidities and a secondary diagnosis of AF, both more common in Aboriginal patients, were strong independent predictors of mortality. Comorbidities contributed substantially to the attenuation of effect in adjusted models.

### AF as a complication

A single study from the USA investigated the influence of ethnicity on the likelihood of AF as a post-operative outcome of coronary artery bypass surgery (Table 3). In this single tertiary referral hospital study, Native Americans constituted only 0.2 % of the total sample of 5823. In crude comparison, new-onset post-operative AF occurred in 18.8 % of Native Americans (mean age 61 years) versus 32.4 % of Causasians (mean age 65) [42].

### AF in clinical groups

Although studies with data on the occurrence of AF in specific clinical groups accounted for the majority of publications identified, AF was not usually the primary research focus (Table 4). Most were conducted in hospital settings and provided crude prevalence data only.

**Table 4:** Studies of frequency of atrial fibrillation in clinical groups

Author (Year) Publication type	Country Indigenous population Calendar period	Methods	Key findings on Indigenous AF	Quality score (Newcastle-Ottawa Scale applied only to Indigenous AF data) Comments
(a) Frequency of atrial fibrillation in primary care consultations				
*Title: Aboriginal and Torres Strait Islander Health Performance Framework 2012 - Detailed Analyses*				
Australian Institute of Health and Welfare (2013) [40] Report	Country: Australia	Design: Cross-sectional	Age-standardised rate (no. of encounters per 1,000 in which AF managed): Indigenous: 15.1 (CI 5.7-24.4) Other: 11.5 (CI 11.0-12.0) Rate ratio 1.3 (NS) Rate difference 3.5 (NS)	NOS (adapted for cross-sectional): 5/10
	Pop: Aboriginal	Data Source: BEACH (written questionnaire, random sample of GPs across Australia)		Likely under-identification of Indigenous patients
	Period: 2006–07 to 2011-12	Setting: General practice attendances		
		Sample size: AF managed during 38 ‘Indigenous’ and 5548 ‘Other’ GP attendances		
(b) Frequency of atrial fibrillation in hospital admissions				
*Title: Atrial fibrillation in Indigenous and non-Indigenous Australians: a cross-sectional study*				
Wong (2014) [29] Conference abstract later published as a journal article	Country: Australia	Design: Retrospective cross-sectional study	Indigenous vs non-Indigenous frequency of AF adjusted for age, sex & CVD comorbidity (odds ratio): 1.183 (CI 0.977-1.432; *p* = 0.085)	NOS (adapted for cross-sectional): 5/10 Unclear definition of AF occurrence (throughout series of ≥1 potential admission per patient) No ‘lookback’ to establish age at 1st AF admission Representativeness of population uncertain from single institution Denominator for comparisons unclear
	Pop: Indigenous Australians (IA)	Data Source: Administrative data	Crude age-stratified frequency of AF Indigenous vs non-Indigenous: <60 yrs 2.57 vs 1.73 % *p* < 0.0001 ≥ 60 yrs 4.61 vs 9.26 % *p* < 0.0001	
	Period: 2000-2009	Setting: Single tertiary hospital (South Australia)	Average age of patients with AF (years): Indigenous 55.4 (SD 13.2) vs Non-Indigenous 74.5 (SD 13.1) *p* < 0.001	
		Sample size: 204668 persons admitted (5892 Indigenous [3.6 %]) 14373 patients with AF diagnosis (221 Indigenous)		
(c) Frequency of atrial fibrillation in specific diagnostic groups				
i. Heart failure				
*Title: Incidence of first heart failure hospitalisation and mortality in Aboriginal and non-Aboriginal patients in Western Australia, 2000-2009*				
Teng (2014) [44] Journal article	Country: Australia	Design: baseline descriptive (within cohort study) hospitalised HF patients	Crude AF prevalence significantly higher in non-Aboriginal patients: 20–55 years	NOS (adapted for cross-sectional): 9/10 15-year clearance period to identify first HF admission; codes validated; 5-year look back for history of AF
	Pop: Aboriginal	Data Source: Linked hospital and death records	Aboriginal = 17.2 % Non-Aboriginal = 26.6 % *p* < 0.001 55–84 years	
	Period: 2000-2009	Setting: Hospital	Aboriginal = 24.6 %% Non-Aboriginal = 44.9 % *p* < 0.001	
		Sample size: 1013 Aboriginal and 16,366 non-Aboriginal hospitalised HF patients		
*Title: Mortality outcomes among status Aboriginals and Whites with Heart Failure*				
Lyons (2014) [43] Journal article	Country: Alberta, Canada	Design: baseline descriptive (within cohort study)	Crude prevalence of AF (as comorbidity): Aboriginals (18 %); Whites (34 %)	NOS (adapted for cross-sectional): 8/10 Albertan Aboriginal population comprises 52 % First Nations, 45 % Métis & 3 % Inuit. Identification of Indigenous status in study based on registration—only First Nations are eligible, of whom 81 % are registered. Métis classified as White in this study.
	Pop: Aboriginal	Data Source: Health care administrative (HMD, ED, ambulatory care) databases linked to the insurance registry (with ethnicity recorded)		
	Period: 2000-2008	Setting: Hospital		
		Sample size: 42,288 whites, 1158 Aboriginals		
ii. Ischaemic heart disease				
*Title: Ischaemic heart disease in New Zealand Māori and non-Māori: an age adjusted incidence in hospitalised patients over 10 years with emphasis on clinical features in the Māori*				
Dancaster (1982) [45] Journal article	Country: NZ	Design: Descriptive	AF detected in 39 % of Māori versus 6 % of non-Māori cases	NOS (adapted for cross-sectional): 3/10 No statistical inference data given for AF proportions Old study—contemporary relevance uncertain
	Pop: Māori	Data Source: Hospital records		
	Period: 1971-1980	Setting: Single regional hospital CCU		
		Sample size: 887 CCU-admitted IHD cases		
iii. Renal failure				
*Title: Atrial fibrillation in haemodialysis patients: do the guidelines for anticoagulation apply?*				
To (2007) [48] Journal article	Country: NZ	Design: baseline descriptive (within cohort study) Data Source: Subjects identified from identified from ANZ Dialysis and Transplant Registry; Hospital records—30 month follow-up	Crude percentage AF: Caucasians 32.8 % Māori 28.6 % Pacific Islanders 19.6 % Asians 16.7 %	NOS (adapted for cross-sectional): 6/10 Underpowered, therefore essentially descriptive study of AF prevalence
	Pop: Māori	Setting: Single hospital haemodialysis unit		
	Period: 2003	Sample size: 155 haemodialysis patients; 28 (18 %) Māori, 51 (33 %) Pacific Islander		
*Title: Trends in the incidence of atrial fibrillation in older patients initiating dialysis in the United States*				
Goldstein (2012) [47] Journal article	Country: US	Design: Cohort study	Crude incidence rate: 148/1000 person-years Compared to non-Hispanic whites, Blacks (−30 %), Asians (−29 %) & Native Americans have lower risk (−42 %) of incident AFCrude incidence rate: 148/1000 person-years Compared to non-Hispanic whites, Blacks (−30 %), Asians (−29 %) & Native Americans have lower risk (−42 %) of incident AF	NOS (cohort): 9/9 Small sample size for Native Americans (1 %).
	Pop: Native Americans	Data Source: US Renal Data System		
	Period: 1995-2007	Setting: Population-based (older Medicare beneficiaries)		
		Sample size: 258,605 (1 % Native Americans)		
*Title: The increasing prevalence of atrial fibrillation among hemodialysis patients*				
Winkelmayer (2011) [46] Journal article	Country: US	Design: series of cross-sectional surveys	Native American HD patients univariate RR for AF 0.38 (vs Causasian); adjusted RR 0.53 (CI 0.50-0.57)	NOS (adapted for cross-sectional): 10/10
	Pop: Native American	Data Source: United States Renal Data System		
	Period: 1992-2006	Setting: maintenance hemodialysis pts—whole of USA		
		Sample size: >10^5^ pts each year of study		
iv. Stroke				
*Title: Prevalence of stroke and coexistent conditions: disparities between Indigenous and non-Indigenous Western Australians*				
Katzenellenbogen (2014) [49] Journal article	Country: Australia	Design: baseline descriptive (within cohort study)	AF more prevalent in Aboriginal than other stroke cases in all age groups <70 years. Crude AF rates were 20 % less in Aboriginal patients due to differing age distributions.	NOS (adapted for cross-sectional): 7/10 (AF not focus of study) Long (20-year) look-back period to identify stroke and AF; AF codes not validated; no stroke type data
	Pop: Aboriginal	Data Source: Linked hospital and death records		
	Period: 2007-2011	Setting: Hospital		
		Sample size: Average 13,591 patients per year (5 % Aboriginal)		
*Title: Racial disparities among Native Hawaiians and Pacific Islanders with intracerebral hemorrhage*				
Nakagawa (2012) [50] Journal article	Country: Hawaii, US	Design: Cross-sectional	Crude prevalence of AF: No significant difference between whites & NHPI (10 % vs 17 %)	NOS (adapted for cross-sectional): 7/10
	Pop: Native Hawaiians & Pacific Islander (NHPI)	Data Source: Clinical database		Single-centre (referral bias). Good clinical data. Limited analysis, given small sample size
	Period: 2004-2010	Setting: Hospital admissions from single tertiary hospital		
		Sample size: 562 ICH cases		
*Title: Disparities among Asians and native Hawaiians and Pacific Islanders with ischemic stroke*				
Nakagawa (2013) [51] Journal article	Country: Hawaii, USA	Design: Cross-sectional	AF prevalence: No significant difference between whites & NHPI Crude prevalence 15 % vs 19 % Adjusted OR 1.06 (0.64-1.75)	NOS (adapted for cross-sectional): 8/10 Single-centre (referral bias). Good clinical data.
	Pop: NHPI	Data Source: Clinical database		
	Period: 2004-2010	Setting: Hospital admissions from single tertiary hospital		
		Sample size: 1,921 ischaemic strokes		
v. Rheumatic heart disease				
*Title: Percutaneous balloon mitral commissurotomy in Indigenous* versus *non-Indigenous Australians*				
McCann (2008) [52] Journal article	Country: Australia	Design: baseline descriptive (within cohort study)	Crude AF prevalence: non-significantly lower in Indigenous Australians (44 % vs 29 %)	NOS (adapted for cross-sectional): 7/10 Only 36 (11 %) of Indigenous Australians. Age-adjusted survival was worse in Indigenous Australians.
	Pop: Indigenous Australians	Data Source: Clinical database		
	Period: 1990-2006	Setting: two tertiary hospitals		
		Sample size: 327		
*Title: A review of valve surgery for rheumatic heart disease in Australia*				
Russell (2014) [53] Journal article	Country: Australia	Design: Cross-sectional	Crude frequency of perioperative AF (%): Indigenous 33.3 Non-Indigenous 41.6 (*p* = 0.039) n.b., difference in mean age: Indigenous 37.4 years Non-Indigenous 65.1 year	NOS: N/A (descriptive study) Comparison of crude frequencies of AF in the two ethnic categories is markedly confounded by age disparity
	Pop: Aboriginal & Torres Strait Islander	Data Source: National Cardiac Surgery Database		
	Period: 2001-2012	Setting: Hospitalised surgery patients		
		Sample size: 1384 RHD (174 Indigenous) compared with 15843 non-RHD valvular surgery patients		
vi. Other cardiac surgery				
*Title: Incidence, secular trends, and outcomes of cardiac surgery in Aboriginal peoples*				
Sood (2013) [54] Journal article	Country: Canada	Design: baseline descriptive (within cohort study)	No significant difference in AF prevalence at baseline (10.1 % non-Aboriginal v 12.0 % Aboriginal)	NOS (cohort): 9/9 Main aims were to compare Aboriginal vs non-Aboriginal patients for incidence, secular trends & outcomes of cardiac surgery. Limited info on AF: crude baseline prevalence in a cohort with known selection bias (demonstrated disparity in selection for surgery)
	Pop: Canadian Aboriginal	Data Source: Provincial Cardiac Surgery registry		
	Period: 1995-2007	Setting: Whole of Manitoba		
	Age: >15 years	Sample size: 12170 (Aboriginal 574; 4.7 %)		
vii. Paediatric patients				
*Title: Excellent cardiac surgical outcomes in paediatric indigenous patients, but follow-up difficulties*				
Rohde (2010) [55] Journal article	Country: Brisbane, AUS	Design: Retrospective review	New atrial arrhythmia as post-surgical complication: 2.4 %	NOS (adapted for cross-sectional): 7/10 Atrial arrhythymia was one endpoint (complication) of follow-up after cardiac surgery.
	Pop: Indigenous Australians (paediatric)	Data Source: Cardiothoracic database, chart review		
	Period: 2002-2009	Setting: Single tertiary hospital		
		Sample size: 112 cases (123 operations)		
*Title: Preoperative risk factors for long-term survival following cardiac surgery for rheumatic heart disease in the young*				
Remenyi (2012) [56] Conference abstract	Country: Auckland, NZ	Design: Retrospective cohort study	Pre-operative AF independently predicted mortality in multivariate analysis (HR 5.2, *p* < 0.01)	NOS: N/A (abstract) No Causasian comparison group
	Pop: Māori & PI	Data Source: Cardiothoracic database, chart review		
	Period: 1990-2006	Setting: Single tertiary hospital		
		Sample size: 212 cases		

*BEACH* Bettering the Evaluation and Care of Health survey, *GP* general practitioner, *NOS* Newcastle-Ottawa Scale, *AF* atrial fibrillation, *CVD* cardiovascular disease, *SD* standard deviation, *HF* heart failure, *HMD* Hospital Morbidity Database, *ED* emergency department, *NZ* New Zealand, *CCU* coronary care unit, *IHD* ischaemic heart disease, *ANZ* Australia & New Zealand, *HD* haemodialysis, *RR* relative risk, *NHPI* Native Hawaiian & Pacific Islander, *N/A*not applicable, *HR* hazard ratio, *PI* Pacific Islander

#### Frequency of atrial fibrillation in primary care consultations

Only a single Australian report provided data on the comparative frequency of AF among Indigenous versus non-Indigenous patients in primary care consultations. The Bettering the Evaluation and Care of Health (BEACH) survey provides written questionnaire data provided by a random sample of general practitioners (GPs) across Australia [40]. In the period 2006–7 to 2011–12, participating GPs reported managing AF during 38 ‘Indigenous’ and 5548 ‘Other’ consultations, reflecting an age-standardised Indigenous:non-Indigenous rate ratio of 1.3 (*p* = NS). Authors of the report suggested that the Indigenous identity of patients had likely been underestimated, making interpretation of the finding problematic.

#### Frequency of atrial fibrillation in hospital admissions

Data on the frequency of AF-coded admissions among hospitalised Indigenous versus non-Indigenous patients were recently published in a large single-institution South Australian study that reported on 204,668 admissions (5,892 Indigenous [3.6 %]) to a tertiary referral centre during the decade 2000–2009 [29]. Indigenous subjects with AF were substantially younger on average than their non-Indigenous counterparts (55.4 years versus 74.5 years). Among admitted patients aged <60 years, the proportion of AF diagnosed was considerably higher among Indigenous patients (2.7 % vs 1.7 %, *p* < 0.0001), while these proportions were reversed in patients ≥60 years (4.61 % vs 9.26 %, *p* < 0.0001). Interpretation of these single institution data is difficult as no population denominator was provided. Furthermore, the (unlinked) data did not allow differentiation of initial and repeat admissions.

#### Frequency of atrial fibrillation in specific diagnostic groups

*(a) Heart failure.* Two cohort studies based on whole-jurisdictional linked administrative data comparing heart failure (HF) in Indigenous and non-Indigenous adult subjects (≥20 years) included AF frequency among the reported baseline characteristics. In a cohort study from Alberta Canada, Lyons *et al.* undertook a study of mortality outcomes among patients hospitalised with incident HF, based on linked inpatient, emergency department, ambulatory care and insurance registry datasets. They reported a crude baseline prevalence of AF as a comorbidity of HF in 18 % in Aboriginal patients versus 34 % in ‘Whites’ (*p* < 0.0001) [43]. Importantly, however, this comparison was confounded by age (baseline mean age [years]: Aboriginal 63; White 75). In a Western Australian cohort study investigating first HF hospitalisation in Aboriginal versus non-Aboriginal patients, baseline crude prevalence of AF was significantly lower among Aboriginal patients in both younger and older age strata (20–54 years: Aboriginal 17.2 %, non-Aboriginal 26.6 % [*p* < 0.001]; 55–84 years: Aboriginal 24.6 %, non-Aboriginal = 44.9 % [*p* < 0.001]) [44].

*(b) Ischaemic heart disease.* No recent studies were found that provided data on AF among Indigenous patients with ischaemic heart disease (IHD). A single descriptive study from NZ investigated the clinical characteristics of patients admitted with IHD to the Coronary Care Unit of a single non-urban hospital during the period 1971–1980 [45]. AF was detected in 39 % of Māori and 6 % of non-Māori cases in crude comparison.

*(c) Renal failure.* Two articles used the United States Renal Data System to report on the epidemiology of AF among dialysis patients with end-stage renal failure. In 2006, the odds ratio for prevalent AF among Native Americans (n = 3332, 1.7 % of total) compared with Caucasian, after multivariate adjustment for age, gender, Medicaid coverage and comorbidities, was 0.55 (CI 0.48-0.63) [46]. Similarly, in longitudinal analysis of older patients (≥67 years) initiating dialysis (total n = 258 605, Native Americans ~1 %), the incidence rate of new AF was 42 % lower among Native American than Caucasian subjects (demographics- and comorbidity-adjusted hazard ratio 0.58 [0.53–0.63]) [47].

Data on baseline AF prevalence were reported from a single hospital haemodialysis unit in Auckland, NZ, in a cohort study investigating the risks and benefits of anticoagulation among haemodialysis patients with AF [48]. The crude AF prevalence was 29 % in Māori (n = 8/28) versus 33 % (19/58) in Caucasian subjects, with no data on age distribution according to ethnicity reported.

*(d) Stroke*. A whole-jurisdictional study of disparities in stroke prevalence between Indigenous and non-Indigenous Western Australians (2007–2011) reported the proportion of stroke patients with a history of AF determined by International Classification of Disease (ICD)-coded hospital diagnoses with 20-year lookback period [49]. The proportion of Indigenous stroke patients with an AF diagnosis was higher in all age-groups below 70 years, although the age-standardised proportions were similar in both ethnicity categories.

In paired cross-sectional studies from Hawaii, racial disparities were investigated among patients admitted with intracerebral haemorrhage (ICH) and those with ischaemic stroke. In patients with ICH (n = 562 cases), the crude frequency of AF among Native Hawaiians categorised together with other Pacific Islanders (NHPI) was 10 %, compared with 12 % in Asians and 17 % in Whites (*p* = NS) [50]. Notably, NHPI with ICH were significantly younger than whites (55 vs 66 years). In patients with ischaemic stroke (n = 1921 cases), there was similarly no significant difference in the crude frequencies of AF at baseline between NHPI and other ethnic groups, but confounding by age was evident [51].

*(e) Rheumatic heart disease.* Two Australian studies provided data on AF among Indigenous subjects with rheumatic heart disease (RHD), but in neither of these was AF an outcome investigated. In a series of 327 patients (36 Indigenous) undergoing percutaneous balloon mitral commissurotomy, the measured difference in AF frequency between the two groups at baseline (44 % non-Indigenous; 29 % Indigenous) was not significant [52]. However, the average age of non-Indigenous subjects was substantially higher (52 versus 36 years). Similarly, a lower crude frequency of AF among Indigenous versus non-Indigenous RHD valvular surgery patients (33.3 % versus 41.6 % *p* = 0.039) reported from an Australia-wide cardiothoracic surgical database (n = 1384 subjects) is difficult to interpret, given the marked age difference between groups [53].

*(f) Other cardiac surgery.* Based on a study using the Manitoba Provincial Cardiac Surgery registry, the crude frequency of AF between Aboriginal and non-Aboriginal aged over 15 years who had undergone cardiovascular surgery during 1995–2007 did not differ significantly (10.1 % versus 12.0 %; *p* = 0.142) [54]. This result did not account for the significant difference in mean age between the two ethnic groups.

*(g) Paediatric AF.* In a descriptive review of outcomes of cardiac surgery at a single tertiary institution in Australia, of all Indigenous paediatric patients (0–17 years) who had cardiac surgery performed in the period 2002–2009 (112 patients, 123 operations), ‘new atrial arrhythmia’ developed as a post-operative in-hospital complication in 2.4 % of cases [55]. A retrospective single-institution study from Auckland, NZ investigating pre-operative determinants of long-term survival following cardiac surgery for RHD was reported in an abstract only. Māori and Pacific Islander children accounted for 98 % of the cohort (n = 212); pre-operative AF was an independent predictor of mortality (hazard ratio [HR] 5.2; *p* < 0.01) [56].

### Health service provision

Only the study from Ontario on the Métis population provided data on health service provision for Indigenous AF, but numbers were small. The crude proportion of patients receiving outpatient echocardiography within 6 months of incident AF diagnosis was 52.9 % in the the Métis (n = 56 incident cases) compared with 42.1 % in the general Ontarian population (*p* = 0.12) [31]. No meaningful data on receipt of evidence-based medications were identified for any of the Indigenous populations.

## Discussion

### Principal findings

Published data on AF in Indigenous populations in the affluent countries included in this review are scanty, fragmentary and of varying quality, with a minority subjected to peer-review. Aside from the potential for real differences in AF epidemiology among Indigenous populations between and within countries, study comparability is limited by differences in quality, design, analytical methods (including means of identifying Indigenous subjects and covariate adjustments), setting (community versus hospital) and calendar time frame.

In consequence, the epidemiology of AF in these populations remains inadequately delineated, with no clear pattern emerging. However, both linked and unlinked administrative hospital data from Canada [28], Australia [29, 30], and NZ [39] suggest that hospitalised AF is more common at younger ages in Indigenous people, with higher first-ever AF hospitalisations in WA (notably under 65 years) and Ontario possibly reflecting disparities in population-based incidence. Concordant with these findings, small studies from Canada and NZ suggest higher AF prevalence among Métis and Māori respectively than the non-Indigenous comparator populations. In contrast, the significantly lower nationwide prevalence of AF among Native Americans than Whites reported in the US study of male military veterans, which incorporated both hospitalisation and ambulatory care data, suggest that higher AF occurrence in Indigenous populations may not apply in the US. Although no difference in short-term (30-day) post-admission mortality was found in the WA cohort, one-year mortality was higher among both WA Aboriginal patients and Ontarian Métis than among the respective comparator populations.

The major antecedents of AF traverse the ‘epidemiological transition’ [57], encompassing sequelae of infectious disease (with streptococcal infection underlying RHD) as well as non-communicable diseases (particularly hypertension and coronary atherothrombosis). As a generalisation, all of these occur in excess and at younger ages among Indigenous peoples, in both North America and Australasia [58–62]. Accordingly, the occurrence of AF would be expected to be correspondingly increased in these populations once age has been accounted for. However, the epidemiological pattern of these disparities is complex, varying among the specific disorders as well as within and between different countries, in magnitude and secular trends. Epidemiological surveillance of Indigenous populations for these underlying disorders remains suboptimal, partly because of inadequate Indigenous identifiers in routinely collected data, particularly in North America.

Unexpectedly, studies of AF frequency among ethnic minority groups such as African Americans, Hispanics and Europeans of African, Afro-Caribbean or Indo-Asian ancestry suggest a ‘racial paradox’, *i.e.*, that the condition is unexpectedly less common among these minority populations than Whites, despite the major risk factors generally being present in excess [63, 64]. The US study of male veterans included in this review suggests that this phenomenon may extend to Native Americans. However, the findings of this study may not be generalisable to the whole US population, insofar as military recruitment is characterised by over-representation of lower socioeconomic strata and so may be disproportionately unrepresentative of the majority ‘White’ population, with under-participation of the most affluent. Recuitment also excludes persons with certain pre-existing health problems (such as congenital or rheumatic cardiac lesions predisposing to AF) and may represent a ‘healthy worker’ effect with those of poorer health not meeting enlistment criteria. Moreover, as outlined in a review of AF and race, ‘[u]nder-ascertainment and differential mortality may partially contribute towards the apparent lower burden of AF [reported] in racial and ethnic minorities’ [63]. No other published data elucidate the relative frequency of AF in the Native American population, despite the substantial research profile of Indigenous CVD in the US, notably that arising from the Strong Heart Study cohort, ‘the largest epidemiologic study of American Indians ever undertaken’ [65].

The available evidence suggests that the ‘paradox’ does not apply to Indigenous minorities in Canada, Australia and NZ and that AF frequency is increased among the Indigenous peoples of these countries, at least at younger ages. However, in interpreting hospitalisation data upon which the largest studies are based, it is important to recognise that frequency of first hospitalisation is not equivalent to incidence rate in a population. Hospitalisations are influenced by health-seeking behaviour and the quality of health care such that disparities between ethnic groups in access to care modify the likelihood of admission. Firstly, inadequate AF management in primary care—including tardy detection—may increase the likelihood of preventable hospitalisation and also skew the spectrum of hospitalised cases towards greater severity, due to poor control of the arrhythmia or associated conditions (such as heart failure) and complications (particularly stroke). Conversely, poor access to hospital care may diminish elective admissions for AF and associated morbidities. In order to establish the true incidence rate of the condition in a population, community-based, longitudinal cohort studies incorporating active surveillance are required. No study of this kind is available for the Indigenous peoples considered.

While a focus on inequities in social determinants and service provision is necessarily paramount in explaining the poorer health of Indigenous peoples, there is a possibility of inter-ethnic variation in genetic predisposition to specific disorders. In relation to AF, a lower prevalence among African Americans compared with Caucasians, despite the more common occurrence of major risk factors in the former, appears to be partly attributable to genetic factors [64, 66]. The extent to which genetic variation modulates AF risk among diverse Indigenous peoples has not been investigated.

Disease outcome indices such as mortality and readmission rates are determined by both disease severity and quality of health service provision during and/or after hospitalisation. Rates for early mortality (30-day mortality among Western Australians following an AF admission and in-hospital mortality among US subjects admitted with AF) were not increased among Indigenous subjects, suggesting that they receive adequate in-hospital care. The data from Western Australia and Ontario reporting relatively high 1-year mortality following an AF hospitalisation could reflect greater severity of disease and/or poorer post-discharge management.

In view of the scanty data, publications were included in this review if they reported Indigenous versus non-Indigenous comparative AF frequency in specific clinical groups. The search results were dominated by these publications. Although several high quality cohort studies were identified, in most cases the principal hypotheses were unrelated to AF, and the AF data were limited to baseline crude frequencies in patient subgroups, including comparisons between Indigenous and other ethnic groups. These data add little to the picture of AF epidemiology in Indigenous populations, except that they demonstrate that AF is characteristically a subsidiary consideration in clinical research, investigated in relation to comorbidities contributing to the clinical complexity of patients rather than examined in its own right. Such data are difficult to interpret, particularly because of the potential for substantial confounding by age and severity of underlying illness. An exception was a stroke prevalence study, showing higher proportions of AF comorbidity in Aboriginal Australians at all ages under 70 years [49]. Of note, age-standardisation did not adequately correct for age differences in that study.

Optimal clinical management of AF is predicated on access to high-quality health care, in relation to which Indigenous populations in general are demonstrably disadvantaged [67, 68]. There is essentially no published evidence on equity of health services provision for AF in these populations although evidence from other cardiometabolic conditions suggests that they are likely to have higher rates of comorbidities that complicate their condition and management [69]. Besides the Ontario paper’s crude comparison of receipt of echocardiography [31], our literature search identified a near absence of data on AF management in Indigenous people. In the broader population, barriers to optimal therapy of AF include underdiagnosis, underestimation of its prognostic severity, inadequate implementation of evidence-based therapeutic guidelines, limited access to care, and inadequate adherence to prescribed treatment [70]. These barriers are likely to be amplified among disadvantaged, marginalised groups such as Indigenous people [71]. AF is especially susceptible to under-diagnosis, given its propensity to be asymptomatic or to manifest with non-specific features, with a substantial proportion of strokes attributable to unrecognised AF [72]. Of particular concern are the many undiagnosed AF patients for whom long-term anticoagulant medication is necessary in order to prevent thromboembolic disease. Notably, since our literature search was performed, a large single-institution South Australian series of patients with known AF (n = 19613 patients; 308 Indigenous) documented that guideline-determined underuse and overuse of anticoagulant medications were both significantly more common (ORs 1.27 and 1.60 respectively) among Indigenous than non-Indigenous patients [73]. In general terms, the socio-economic and environmental factors predisposing Indigenous peoples to conditions associated with AF are also likely to compromise its clinical management [74].

### Strengths and limitations

This scoping review contributes to a genre that brings together research on the health of Indigenous minorities living in affluent countries [8, 75–78]. Its strengths include an exhaustive search of established databases, efforts to access the grey literature and appraisal of study quality to provide an overview of existing research into AF in Indigenous peoples of the selected countries. The principal limitation of the review was the need to frame its design as a scoping study without a focussed research question, based on anticipation of scanty published data. Furthermore, there are unavoidable trade-offs in the exhaustive approach to literature searching that extends beyond indexed academic databases. Although Google Scholar allows searching the full text of publications, thereby allowing recognition of data that may not be identified through subject headings keywords, titles and abstracts, this interface has less sophisticated functionality for systematic searching than traditional databases [79]. Furthermore, the ‘grey’ literature such as government websites is characterised by inconsistent quality by virtue of the absence of peer review, as well as poor searchability given the diversity of formats, the absence of controlled vocabulary indexing, and sheer volume [80]. The NOS used to appraise the quality of studies of non-randomised design is limited by subjectivity, as demonstrated by high inter-observer variation [81].

## Conclusions

Accurate epidemiological data are a prerequisite to optimising the equity and efficiency of service provision for CVD among Indigenous people. AF is an eminently treatable condition. Timely detection of AF is necessary, firstly to maximise the chances of reversing to normal sinus rhythm if practicable, usually accompanied by addressing the underlying cause, and secondly to institute pharmacological management to reduce to the likelihood of potentially life-threatening complications. Relative underdetection and consequent skewing of hospital presentations are obstacles to quantifying the comparative frequency of AF in these populations satisfactorily [30, 63]. Furthermore, the reduced life expectancies of Indigenous populations alter the age structure, requiring cautious interpretation of disparities in rates: conventional age standardisation and age-adjustments may not adequately control for age differences between Indigenous and non-Indigenous populations [49]. The small numbers of people belonging to Indigenous minorities also create challenges in making meaningful rate comparisons between groups within small age bands.

It is crucial that health systems in jurisdictions with Indigenous minorities incorporate data on Indigenous identity in routinely collected administrative health surveillance data from primary health care and hospitals [82, 83]. Optimally, sophisticated data linkage systems are needed to facilitate person-based (rather than merely event-based) surveillance measures, in order to identify incident cases, distinguish re-presentations/re-admissions and determine outcomes. The inclusion of AF in the conditions reported in government publications could begin to elucidate existing disparities and increase attention on the need for effective interventions. Additionally, there is an urgent need for sufficiently powered, community-based studies of AF epidemiology in diverse Indigenous populations that incorporate active case-finding and have a valid comparator population.

## Additional file

Additional file 1: Table S1. Search terms 19 May 2014 (PubMed version) (DOCX 23 kb)

## Abbreviations

AF: Atrial fibrillation
ASRR: Age-standardised rate ratio
BEACH: Bettering the Evaluation and Care of Health
BMI: Body- mass index
CI: Confidence interval
CVD: Cardiovascular disease
ED: Emergency department
GP: General practitioner
HF: Heart failure
HR: Hazard ratio
ICD: International Classification of Disease
ICH: Intracranial haemorrhage
IHD: Ischaemic heart disease
NHPI: Native Hawaiian and Pacific Islander
NOS: Newcastle-Ottawa Scale
NZ: New Zealand
RHD: Rheumatic heart disease
US: United States
WA: Western Australia
