# Supplementary material for: Atrial fibrillation in the Indigenous populations of Australia, Canada, New Zealand, and the United States: a systematic scoping review
Source: BMC Cardiovasc Disord. 2015 Aug 13;15:87. doi: 10.1186/s12872-015-0081-6 (PMC4535416; doi:10.1186/s12872-015-0081-6)
Supplement: Additional file 1: Table S1. — Search terms 19 May 2014 (PubMed version) (DOCX 23 kb) [file 12872_2015_81_MOESM1_ESM.docx]

Table S1: Search terms 19 May 2014 (PubMed version)

**‘Indigenous’ terms:**

health services, indigenous [mh]

oceanic ancestry group [mh]

american native continental ancestry group [mh]

indigenous

indigene*

aborigin*

“torres strait islander”

maori*

polynesian*

“native american”

“american indian”

amerind*

alaskan*

eskimo*

“native hawaiian”

“first nation”

inuit*

metis

“native canadian”

“canadian indian”

**‘Atrial fibrillation’ terms:**

"atrial fibrillation" (*subsuming* atrial fibrillation [mh])

"auricular fibrillation"

"atrial flutter"
